# Reactive Hyperemia Reveals Fractal Scaling and Multiscale Complexity in Photoplethysmography Waveforms

**DOI:** 10.3390/biology15131073

**Published:** 2026-07-04

**Authors:** Henrique Silva

**Affiliations:** 1 Research Institute for Medicines (iMed.ULisboa), Faculty of Pharmacy, Universidade de Lisboa, Av. Prof. Gama Pinto, 1649-003 Lisbon, Portugal; henrique.silva@edu.ulisboa.pt; 2 Department of Pharmacy, Pharmacology and Health Technologies, Faculty of Pharmacy, Universidade de Lisboa, Av. Prof. Gama Pinto, 1649-003 Lisbon, Portugal; 3 Biophysics and Biomedical Engineering Institute (IBEB), Faculty of Sciences, Universidade de Lisboa, Campo Grande, 1749-016 Lisbon, Portugal

**Keywords:** post-occlusive reactive hyperemia, fractality, detrended fluctuation analysis, signal complexity, multiscale entropy analysis

## Abstract

Post-occlusive reactive hyperemia (PORH) is a classical probe of microvascular function, yet its assessment remains largely based on amplitude-derived indices that do not capture the temporal organization of vascular regulation. Photoplethysmography (PPG), widely used in clinical and wearable technologies, offers a practical platform for nonlinear characterization of PORH. Twelve healthy adults underwent a standardized PORH protocol (10 min baseline, 5 min suprasystolic occlusion, 10 min reperfusion) with bilateral reflective green-light PPG. Pulse amplitude, detrended fluctuation analysis (global DFA α exponent), and multiscale entropy (Complexity Index, CI) were computed in 5 min epochs. Occlusion nearly abolished pulsatility in the test limb but produced only modest changes in fractal structure, as α decreased minimally despite near-zero flow. In contrast, CI showed a marked collapse, indicating loss of multiscale organization. During reperfusion, α exhibited a trend toward increased fractal persistence, whereas CI recovered only partially. Contralateral responses were small and detectable mainly through subtle reductions in α during occlusion and consistently higher CI compared with the test limb. These findings indicate that occlusion disrupts multiscale complexity without eliminating fractal persistence, whereas reperfusion restores correlation structure and only partially re-establishes dynamical richness. Overall, DFA and MSE reveal nonlinear features of PORH that are not captured by conventional amplitude-based metrics, extending the physiological interpretation of microvascular responses using widely available PPG technology.

## Simple Summary

Reactive hyperemia, the transient increase in blood flow following the release of arterial occlusion, is traditionally assessed by changes in blood flow/volume. Although informative, this metric does not capture how microvascular signals are temporally organized. In this study, bilateral photoplethysmography (PPG) recordings were analyzed using detrended fluctuation analysis and multiscale entropy analysis to quantify fractal structure and dynamical complexity during the different phases of an arterial occlusion procedure. Occlusion markedly reduced pulsatility in the test limb, yet fractal persistence remained relatively stable, whereas multiscale complexity was substantially diminished. Upon reperfusion, long-range correlations were strengthened and complexity partially recovered. These nonlinear features reveal regulatory aspects that are not detectable with amplitude-based indices alone. They also highlight the potential of PPG to capture previously inaccessible microvascular dynamics.

## 1. Introduction

Post-occlusive reactive hyperemia (PORH) is a classical functional test of microvascular health and is widely regarded as an indirect marker of endothelial integrity [1]. Although often interpreted as a predominantly endothelial phenomenon, PORH arises from the coordinated interaction of several other determinants, including sensory nerve activity, myogenic responses, and local endothelial mechanisms, whose relative contributions vary across vascular beds and methodological settings. PORH has been assessed using diverse optical and hemodynamic techniques such as laser Doppler flowmetry (LDF), laser speckle contrast imaging (LSCI), arterial tonometry, near-infrared spectroscopy, photoplethysmography (PPG), and videocapillaroscopy [2,3,4,5,6,7]. Conventional analytical approaches rely on simple temporal descriptors, including peak hyperemia, time to peak, and area under the reperfusion curve. While clinically useful, these indices provide only a coarse representation of the underlying regulatory dynamics and do not capture higher-order temporal organization such as complexity or fractal structure. In PPG-based assessments of PORH, this paradigm is largely preserved, with most studies focusing on amplitude-derived metrics and basic time-domain descriptors, occasionally complemented by waveform morphology analysis to characterize the occlusion–reperfusion response [8,9,10,11]. While these approaches capture the magnitude and timing of the hyperemic response, they largely neglect the temporal organization and multiscale structure of microvascular fluctuations. More recently, there has been growing interest in extracting additional physiological information from PPG signals using advanced signal processing techniques, highlighting the potential of PPG to provide physiological information beyond conventional amplitude-based analysis.

Photoplethysmography signals are intrinsically rich and multiscaled, reflecting cardiac pulsatility, respiratory modulation, myogenic vasomotion, fluctuations in sympathetic vasoconstrictor tone, and endothelial rhythmicity, each operating within its own characteristic frequency range [8,9,10]. The superposition of these oscillatory components gives rise to complex temporal dynamics spanning multiple time scales. In the microcirculation, these oscillatory components are collectively referred to as flowmotion. The myogenic component [~0.1 Hz] is driven by pressure–flow autoregulation, the neurogenic component [~0.04 Hz] primarily reflects sympathetic vasoconstrictor tone, and the endothelial components [~0.01–0.005 Hz] are associated with nitric oxide-dependent and -independent vasodilation [10,11,12,13]. These low-frequency components modulate tissue perfusion and reorganize dynamically during PORH. Quantifying how these scales reorganize during physiological perturbations such as PORH offers insight into the mechanisms governing microvascular perfusion [14,15]. Because PPG captures their integrated expression, it is particularly well suited for nonlinear analysis of microvascular dynamics.

A wide range of physiological time series, including electrocardiography, arterial blood pressure, and microvascular blood flow, exhibit fractal properties and long-range temporal correlations, which are hallmarks of adaptive cardiovascular control [14,15,16,17,18,19]. Detrended fluctuation analysis (DFA) provides a robust measure of fractal persistence, which changes in both disease and acute physiological challenges [20,21,22,23,24,25,26,27,28]. In parallel, signal complexity, commonly assessed with multiscale entropy [MSE], reflects the richness and unpredictability of variability across scales; healthy physiological systems tend to exhibit high complexity, whereas constrained or pathological states often display marked reductions [29,30,31,32]. Although DFA and MSE have previously been applied to LDF PORH recordings [25], their combined use has not yet been explored in the context of PPG-derived PORH, despite PPG being one of the most widely available and clinically relevant optical techniques for microvascular assessment [33,34]. This gap is relevant because PPG is the most widely deployed optical technology in both clinical and consumer-grade devices, making its nonlinear characterization especially attractive for scalable microvascular assessment.

The aim of the present study was therefore to characterize the fractal scaling behavior and multiscale complexity of PPG signals during a standard PORH protocol, both in the occluded limb and in the contralateral limb. By integrating DFA and MSE, we sought to uncover dynamical signatures of microvascular reactivity that remain invisible to traditional amplitude-based metrics.

## 2. Materials and Methods

### 2.1. Participants

Twelve healthy young adults (21.7 ± 1.9 y.o.; 6 females) were recruited from the local academic community following the same inclusion/exclusion criteria and pre-experimental preparation previously described by Silva et al. (2025) [35]. All participants gave written informed consent. In brief, participants were non-smokers, reported no cardiovascular, neurological or psychiatric disorders, were not taking regular medication, and female subjects had regular menstrual cycles without hormonal contraception. Standardized preparation (abstinence from caffeine and exercise for 12 h, light fasting, and bladder emptying before instrumentation) was used to minimize autonomic and endothelial variability. This study was conceived as an exploratory proof-of-concept investigation. Because no previous study had applied combined DFA and MSE analysis to PPG-derived PORH recordings, reliable effect-size estimates were unavailable and a formal a priori sample-size calculation could not be per-formed. A repeated-measures within-subject design was adopted, with each participant serving as their own control across baseline, occlusion, and recovery phases, thereby reducing inter-individual variability. The sample size was comparable to previous physiological studies employing nonlinear analyses of microvascular signals. The study was approved by the institutional ethics committee (11/2024) and complied with the Declaration of Helsinki [36].

### 2.2. Procedure

Participants underwent a standard suprasystolic arterial occlusion on a randomly selected arm (test). The occluded arm was randomly assigned to avoid systematic bias related to laterality or handedness. Participants were given a 20 min acclimation period prior to instrumentation to ensure thermal and autonomic stabilization. The protocol, performed at rest, consisted of a continuous 25 min recording divided into three predefined epochs: a 10 min baseline period, a 5 min arterial occlusion, and a 10 min post-occlusion hyperemic phase. To induce occlusion, a pneumatic cuff placed around the arm was rapidly inflated to 200 mmHg (within approximately 5 s) and maintained at this pressure for the entire 5 min interval. At the end of the occlusion period, the cuff was rapidly released (also within ~5 s) to elicit reactive hyperemia. The contralateral limb (control) remained unmoved during the entire procedure. All sessions were conducted in a controlled laboratory environment with stable temperature and humidity (22–24 °C; 40–65%).

### 2.3. Technologies

Photoplethysmography signals were recorded using reflective green light PPG sensors (530 nm wavelength) placed on the distal phalanges of both index fingers. These sensors provide a measure of the blood volume pulse through changes in backscattered light [33,34]. Skin blood flow was estimated from pulse amplitude, defined as the difference between the systolic peak of each pulse and the onset of the subsequent pulse wave, and expressed in arbitrary units (AU). While pulse amplitude is widely used as a surrogate of peripheral perfusion in PPG studies, it represents an indirect proxy of blood flow and may be influenced by factors such as local vascular compliance, tissue optical properties, and sensor contact pressure. In the present study, sensor placement was standardized using adhesive fixation without external compression, and recordings were performed under controlled resting conditions to minimize motion-related artifacts. These limitations primarily affect absolute amplitude values; however, they are unlikely to substantially alter the temporal structure of the signal, which underlies the nonlinear analyses performed in the present study.

All sensors were connected to a BITalino (r)evolution Plugged board (PLUX Biosignals, Lisbon, Portugal). Signals were sampled at 100 Hz and acquired using the OpenSignals (r)evolution software (PLUX Biosignals, Lisbon, Portugal). Data were subsequently exported and processed in MATLAB R2015a (MathWorks, Natick, MA, USA) for further analysis.

### 2.4. Detrended Fluctuation Analysis

Detrended fluctuation analysis (DFA) was applied to quantify long-range temporal correlations in the PPG-derived pulse amplitude time series described in Section 2.3. Prior to nonlinear analysis, no additional filtering or interpolation was applied to the PPG-derived pulse amplitude time series, in order to preserve its intrinsic temporal structure. The PPG-derived pulse amplitude time series was downsampled to 10 Hz to reduce cardiac-cycle dominance while preserving low-frequency vasomotor components relevant to microvascular regulation. DFA provides a scale-dependent estimate of fractal persistence (α, alpha exponent), reflecting the degree to which fluctuations remain correlated across progressively larger temporal windows [37].

Each 25 min recording was segmented into three 5 min epochs corresponding to baseline (5–10 min), occlusion (10–15 min), and post-occlusion hyperemia (15–20 min). Five-minute epochs are commonly used in PORH studies and provide sufficiently quasi-stationary segments for DFA while excluding the highly nonstationary periods associated with cuff inflation and deflation. DFA was performed separately for each epoch and for each limb. The algorithm followed the standard procedure described in Peng et al. (1995) [37]. Briefly, for a given segment *x*(*i*), the cumulative profile

$$Y\left(k\right)=\sum_{i=1}^{k}\left(x\left(i\right)-\bar{x}\right)$$

was computed and divided into non-overlapping windows of equal length *n*. In each window, a least-squares linear fit (DFA-1) was used to remove local trends, and the root-mean-square fluctuation *F*(*n*) of the detrended profile was calculated. DFA-1 was chosen because higher-order detrending can distort the low-frequency vasomotor oscillations that contribute to α and is discouraged for short biological segments. This procedure was repeated across a range of window sizes. To ensure robust estimation in 5 min segments, scaling windows were initially defined over a theoretical range of *n* = 10–1000 samples (1–100 s at 10 Hz). However, due to the finite length of each segment, windows above approximately *n* = 600 provide fewer than five independent realizations and therefore do not yield stable fluctuation estimates. Only scales meeting this criterion were included in the log–log regression, ensuring that *α* reflects vasomotor and autoregulatory dynamics rather than cardiac or respiratory components. The relationship between fluctuation magnitude and window size

$$F\left(n\right)\sim n^{\alpha }$$

was then obtained by linear regression of log *F*(*n*) versus log *n*. The slope of this regression provided the global scaling exponent *α*, interpreted as follows: *α* ≈ 0.5 indicates uncorrelated (white-noise-like) behavior, *α* > 0.5 reflects persistent long-range correlations, and *α* < 0.5 indicates anti-persistence. Given the short 5 min duration of each epoch, the scaling range is insufficient to reliably identify separate short- and long-term scaling regions or to resolve genuine crossover points. Apparent crossovers in microvascular signals of this length are typically artefactual, arising from scale truncation rather than true multiscaling. For this reason, only the global α exponent, estimated over the full valid range, was retained, as it provides the most stable and physiologically interpretable estimate for short biological recordings. No interpolation or additional filtering was applied to the downsampled PPG amplitude time series, in order to preserve the intrinsic temporal structure of the signal. This approach minimizes the risk of artificially altering correlation properties and entropy estimates, which are sensitive to preprocessing procedures. DFA computations were implemented in MATLAB 2015a (MathWorks, Natick, MA, USA) using a custom-validated script based on the original Peng et al. formulation [37].

### 2.5. Multiscale Entropy Analysis

Multiscale entropy (MSE) analysis was used to quantify the dynamical complexity of the PPG signal across multiple temporal scales [29]. MSE evaluates how the regularity of a time series evolves after coarse-graining, providing insight into the richness and adaptability of microvascular control mechanisms. The PPG-derived pulse amplitude time series, sampled at 100 Hz, was analyzed in the same 5 min epochs defined for DFA (baseline: 5–10 min; occlusion: 10–15 min; hyperemia: 15–20 min). No downsampling was applied in order to preserve high-frequency components that contribute to microvascular variability. As a consequence, short-scale entropy values may reflect contributions from higher-frequency components, including cardiac and respiratory activity. However, the multiscale framework captures the integrated structure of the signal across temporal domains, allowing meaningful characterization of microvascular dynamics. For each scale factor *τ*, the coarse-grained time series *y*^(*τ*)^ was constructed by averaging non-overlapping segments of length *τ*, using the standard floor rule to avoid interpolation artifacts and to ensure strict comparability across scales:

$$y^{\left(\tau \right)}\left(j\right)=\frac{1}{\tau }\sum_{i=\left(j-1\right)\tau +1}^{j\tau }x\left(i\right),\;\;j=1,\ldots ,\ldots \left[\begin{array}{c} N\\ \tau \end{array}\right]$$

where *x*(*i*) is the PPG-derived pulse amplitude time series and *N* its length. Scales *τ* = 1–40 were used, corresponding to effective temporal resolutions from 10 to 400 ms. Sample entropy (SampEn) was computed for each coarse-grained series using embedding dimension *m* = 2 and tolerance *r* = 0.15. This parameter set follows established recommendations for short physiological recordings: *m* = 2 provides stable entropy estimates in 5 min segments and avoids undefined values due to insufficient template matches, whereas *r* = 0.15 balances robustness against noise with sensitivity to physiologically meaningful structure. Higher *m* or smaller *r* values produce sparse matches in short microvascular recordings and lead to unstable entropy estimates. Given a sequence *y*^(*τ*)^, SampEn was defined as:

$$\mathrm{SampEn}(m,r)=-ln(\frac{A}{B})$$

where B is the number of pairs of embedded vectors of length *m* that match within tolerance *r*, A is the number of pairs that remain matched when extended to length *m* + 1. Lower SampEn indicates greater predictability (lower complexity), whereas higher SampEn reflects richer, more irregular dynamics.

Overall multiscale complexity was quantified by the Complexity Index (*CI*):

$$CI=\sum_{\tau =1}^{40}{SampEn}_{\tau },$$

providing an integrated measure of variability across all examined time scales. Higher *CI* values indicate greater multiscale complexity and more adaptive physiological dynamics, whereas lower *CI* values reflect more regular or constrained temporal organization across scales. MSE calculations were performed in MATLAB using a custom wrapper around a fast C-based implementation (mse.exe), ensuring consistent coarse-graining and entropy computation across participants.

### 2.6. Statistical Analysis

All statistical analyses were performed using IBM SPSS Statistics v21 (IBM Corp., Armonk, NY, USA). Given the small sample size (N = 12) and the non-normal distribution of perfusion, fractal- and entropy-derived metrics, all results are reported as median and interquartile range (IQR). Comparisons across the three experimental phases (baseline, occlusion, and hyperemia) were conducted using the Wilcoxon signed-rank test for paired samples. Phase-to-phase comparisons were performed separately for each limb (test and contralateral) to distinguish local from systemic contributions to microvascular dynamics. To evaluate bilateral differences within each phase (test limb vs. contralateral limb), additional Wilcoxon signed-rank tests were performed, accounting for the inherent within-subject dependency of matched measurements. Statistical significance was defined as *p* < 0.05.

## 3. Results

**Figure 1:**
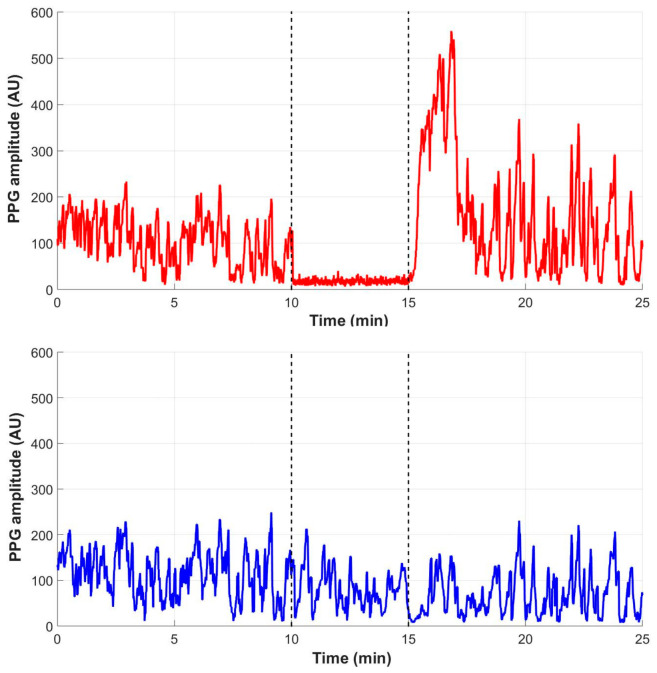
Photoplethysmography (PPG) amplitude traces for the test limb (**upper**, red) and contralateral limb (**lower**, blue) in a representative participant (Subject #8, male, 23 y.o.).

**Figure 2:**
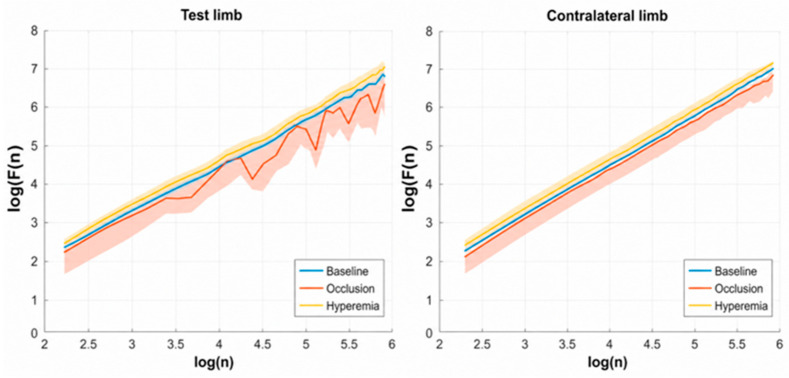
Detrended fluctuation analysis (DFA) curves (median and interquartile range) across all protocol phases for the test limb (**left**) and contralateral limb (**right**). Solid lines represent the median DFA values, and the shaded areas indicate the interquartile range (IQR).

**Figure 3:**
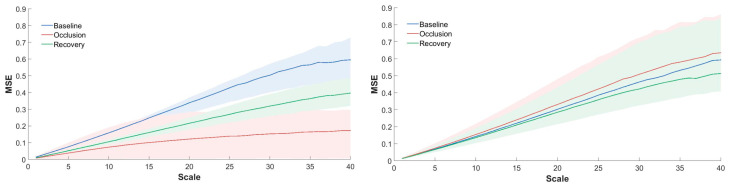
Multiscale entropy (MSE) curves (median and interquartile range) across all protocol phases for the test limb (**left**) and the contralateral limb (**right**). Solid lines represent the median MSE values, and the shaded areas indicate the interquartile range (IQR).

The PORH protocol induced the expected physiological response in the test limb, validating the experimental model used for nonlinear analysis. Participant characteristics are summarized in Table 1. Median and IQR values for all DFA and MSE parameters across the baseline, occlusion, and recovery phases are presented in Table 2. Figure 1 provides the representative temporal evolution of the PPG signal during the PORH protocol, whereas Figure 2 and Figure 3 illustrate the distributions of the corresponding DFA and MSE metrics. 

In the test limb, pulse amplitude demonstrated the expected strong phase dependency. Median amplitude dropped sharply from 434 AU at baseline to 13 AU during occlusion (*p* = 0.003), confirming near-complete suppression of distal pulsatility. During recovery, amplitude increased to 509 AU, a robust hyperemic rebound that approached, but did not reach, statistical significance relative to baseline (*p* = 0.084). In the contralateral limb, amplitude remained within a stable physiological range across phases, with median values of 462, 457, and 496 AU at baseline, occlusion, and recovery, respectively. Only the decrease from baseline to occlusion was significant (*p* = 0.006), reflecting a modest systemic or autonomic influence of unilateral arterial occlusion. Between-limb comparisons showed no differences at baseline (*p* = 0.158), significantly lower test-limb amplitude during occlusion (*p* = 0.002), and a trend toward higher amplitude in the test limb during recovery (*p* = 0.084), consistent with localized reperfusion hyperemia.

In the test limb, the global fractal exponent showed only modest phase-dependent modulation. Alpha decreased slightly from baseline to occlusion, representing the only statistically significant within-limb change (*p* = 0.015). During recovery, alpha returned to baseline-like values, indicating that fractal dynamics were only transiently affected by arterial occlusion. In the contralateral limb, alpha remained stable across all phases, with no significant within-limb differences. This pattern is consistent with the absence of substantial systemic or centrally mediated changes in fractal dynamics during unilateral occlusion. Between-limb comparisons revealed a significant difference during the occlusion phase (*p* = 0.007), whereas no differences were observed at baseline or during recovery. Overall, DFA revealed only a modest, transient reduction in fractal scaling during arterial occlusion, with no evidence of sustained reorganization during reactive hyperemia. MSE analysis revealed a distinct signature of dynamical reorganization. In the test limb, the CI dropped sharply from 13.4 at baseline to 4.5 during occlusion (*p* = 0.006). During recovery, CI increased to 8.6 but remained significantly lower than baseline (*p* = 0.002), indicating only partial restoration of multiscale structure. In the contralateral limb, CI remained relatively stable across phases, varying only modestly from 12.3 at baseline to 13.5 during occlusion and 11.4 during recovery, with no significant within-limb changes. However, limb comparisons showed significantly higher complexity in the contralateral limb during both occlusion (*p* = 0.004) and recovery (*p* = 0.002), confirming that the suppression of multiscale dynamics was strictly localized to the occluded microvascular bed.

## 4. Discussion

To the author’s knowledge, this is the first study to assess DFA and MSE metrics from PORH recordings made with PPG, demonstrating that nonlinear signatures of hyperemia can be extracted from an inexpensive and widely available optical technology. The present study focused on the acute nonlinear dynamics elicited during a single PORH session. Consequently, the between-session reproducibility of DFA- and MSE-derived metrics was not evaluated and should be addressed in future studies. Although PORH is traditionally quantified using amplitude-based metrics, our findings show that DFA and MSE reveal regulatory attributes that remain inaccessible to conventional descriptors of the reperfusion curve.

DFA and MSE have been widely used to characterize the temporal organization of cardiovascular variability. Originally introduced to quantify long-range correlations in heartbeat intervals, DFA was later applied to arterial blood pressure, respiratory activity and cerebral blood flow oscillations [19,20,21,22,23,24,28], consistently showing that healthy physiological systems exhibit fractal 1/f-type dynamics. Likewise, entropy-based approaches have been increasingly recognized as useful for characterizing the complexity of cardiovascular signals across physiological and pathological conditions, reinforcing the relevance of MSE as a marker of dynamical organization [29,30]. In particular, MSE has been used to assess the multiscale richness of electrocardiography, arterial blood pressure and microvascular recordings, and to detect complexity loss in conditions such as aging, diabetes and sepsis [38,39,40,41]. In the microcirculation, DFA and MSE have been applied chiefly to laser Doppler and near-infrared spectroscopy signals, and more recently to PPG during local and neural hemodynamic responses [23,24,25]. However, despite this broad use, no prior work has examined fractal or multiscale entropy signatures during PPG-derived PORH, even though PPG is ideally suited for low-frequency dynamical analyses and widely available in clinical and consumer devices.

From a physiological perspective, DFA and MSE capture complementary aspects of signal dynamics. The DFA alpha exponent quantifies the degree of temporal correlation across scales, reflecting how strongly current fluctuations depend on past behavior. In microvascular signals, persistent scaling (α > 0.5) is typically associated with coordinated vasomotor activity arising from the interaction of endothelial, neurogenic, and myogenic mechanisms operating over multiple time scales. In contrast, MSE assesses the irregularity of the signal after progressive coarse-graining, providing a measure of the richness of multiscale fluctuations. Higher entropy values indicate a more complex and adaptable system, in which multiple regulatory processes interact dynamically, whereas lower entropy reflects a more constrained and predictable state. Together, these metrics provide insight into both the correlation structure and the multiscale organization of microvascular regulation.

The relative stability of α despite profound reductions in pulse amplitude highlights that DFA primarily reflects temporal organization rather than signal magnitude. Importantly, this property allows DFA estimates to remain relatively stable even under conditions of markedly reduced physiological activity, particularly in short data segments such as those used in the present study. In addition, the short 5 min epochs limit the ability to resolve distinct short- and long-term scaling behaviors, reinforcing the use of a global alpha exponent. Within this methodological context, the absence of significant alpha changes during occlusion is therefore an expected consequence of the underlying physiology and the finite data length, rather than evidence against true microvascular suppression. In this context, the alpha values observed here are consistent with those reported in LDF-based studies of microvascular flow, which typically show persistent scaling behavior even under perturbed or low-flow conditions.During reperfusion, the alpha exponent showed a non-significant trend toward increased fractal persistence. Although this change did not reach statistical significance, its direction is consistent with previous observations obtained from LDF-based PORH recordings [25]. These findings suggest that reperfusion may be accompanied by a partial restoration of temporal organization in microvascular dynamics, although this interpretation should be considered preliminary given the exploratory nature of the study.

Multiscale entropy exhibited a complementary pattern. The marked reduction in CI during occlusion indicates a substantial loss of multiscale complexity when perfusion is markedly reduced. Conversely, the partial recovery of CI during reperfusion suggests a restoration of signal complexity following the release of arterial occlusion. These findings are consistent with the broader concept that physiological systems exhibit greater dynamical complexity under normal conditions, whereas flow restriction is associated with more predictable and less complex signal behavior [42]. However, the incomplete recovery of CI observed during the reperfusion phase should be interpreted cautiously, as the present study was not designed to identify the specific physiological mechanisms underlying these changes.

Contralateral responses were modest. Although small fluctuations in alpha were observed, neither DFA nor MSE demonstrated significant phase-dependent changes in the contralateral limb. This finding suggests that the nonlinear alterations detected during PORH were predominantly localized to the occluded limb. The preservation of complexity in the contralateral recordings, together with the limited changes in fractal scaling, further supports the notion that DFA and MSE capture distinct aspects of signal organization. Future studies incorporating direct measures of autonomic and hemodynamic regulation will be required to determine whether subtle contralateral nonlinear responses accompany unilateral arterial occlusion.

Together, DFA and MSE revealed complementary aspects of PPG dynamics during PORH. DFA characterized the persistence of temporal correlations, whereas MSE quantified the richness of multiscale variability throughout the occlusion–reperfusion sequence. These findings support the view that nonlinear analyses provide information that is not fully captured by conventional amplitude-based metrics. Importantly, the present results demonstrate the feasibility of applying DFA and MSE to PPG-derived PORH recordings using a widely available and inexpensive technology. Future studies in larger cohorts and clinical populations will be required to determine the physiological and potential clinical significance of these nonlinear markers.

An important aspect of the present work is the use of PPG as a platform for nonlinear characterization of reactive hyperemia. Unlike techniques such as LDF or LSCI, PPG is inexpensive, widely available, and already incorporated into many clinical and wearable devices. The present findings demonstrate the feasibility of applying DFA and MSE to PPG-derived PORH recordings and support further investigation of these approaches in larger studies and clinical populations.

## 5. Limitations

This study has several limitations. First, the sample size was relatively small, which may restrict the generalizability of the results and reduce statistical power for detecting more subtle contralateral or phase-dependent effects. Second, although the use of 5 min segments is consistent with standard PORH methodology, such short windows inherently limit the upper range of reliable temporal scales, particularly for multiscale entropy and for the identification of distinct short- and long-term slopes in DFA. Consequently, only the global DFA exponent was analyzed, since crossover estimation was unstable in these short segments. Third, the 10 min reperfusion period may not have been sufficient to fully capture the reemergence of multiscale complexity, which may continue to evolve beyond the timeframe examined here. Fourth, although downsampling reduces cardiac dominance, residual respiratory influences may still affect short-scale correlations in DFA. Finally, although the occluded limb was randomly assigned, potential effects of lateralization or handedness were not specifically investigated and may represent an additional source of variability in microvascular responses. Future studies using longer recordings, assessing multiple vascular beds, and including clinical populations will be essential to confirm the robustness, physiological interpretability, and translational potential of these nonlinear metrics.

## 6. Conclusions

In summary, this study demonstrates that detrended fluctuation analysis and multiscale entropy provide complementary and physiologically meaningful insights into the temporal organization of PPG signals during PORH. Arterial occlusion induced a marked collapse of multiscale complexity, while reperfusion was characterized by strong fractal persistence and partial restoration of dynamical richness, features that remain undetected by conventional amplitude-based PORH metrics. Contralateral effects were modest but consistent with mild autonomic spillover. Together, these findings highlight the value of nonlinear analysis as a powerful extension of traditional PPG-based vascular assessment. DFA and MSE reveal non-redundant aspects of microvascular reactivity and offer a richer framework for characterizing vascular responsiveness in health and disease. Future work in clinical populations and with longer recordings will determine the translational potential of these metrics as sensitive markers of endothelial and microvascular function. Given the widespread availability of PPG in both clinical and consumer devices, these nonlinear markers could be readily implemented in routine vascular screening and autonomic function testing.

## Figures and Tables

**Table 1:** Individual participant characteristics and baseline hemodynamic measurements (N = 12). SBP—systolic blood pressure; DBP—diastolic blood pressure.

ID	Sex	Age	Test Limb	BMI (kg/m^2^)	SBP (mmHg)	DBP (mmHg)	Pulse (bpm)	PPG Amplitude	
								Test	Control
#1	Female	26 y.o.	right	24.4	108	75	75	553	639
#2	Female	20 y.o.	right	19.8	113	81	70	369	85
#3	Female	23 y.o.	right	23.7	113	83	80	206	361
#4	Female	20 y.o.	left	18.3	105	70	71	822	716
#5	Female	20 y.o.	left	23.8	105	67	65	518	562
#6	Female	23 y.o.	right	22.6	133	93	63	23	36
#7	Male	21 y.o.	left	21.7	121	87	63	628	661
#8	Male	23 y.o.	right	24.9	125	86	78	173	230
#9	Male	20 y.o.	left	21.9	120	75	65	75	96
#10	Male	23 y.o.	left	25.4	111	64	53	674	692
#11	Male	21 y.o.	right	19.7	122	74	73	252	257
#12	Male	20 y.o.	left	19.4	116	69	66	631	776

**Table 2:** Median and interquartile range (IQR) values for PPG amplitude, DFA alpha exponent, and MSE Complexity Index across all experimental phases and limbs (N = 12). Within-limb phase comparisons were performed between baseline and occlusion and between baseline and recovery using the Wilcoxon signed-rank test. Between-limb comparisons were performed separately for each phase. (* *p* < 0.05).

Parameter	Limb	Test Limb			Limb Comparison (T vs. C)		
		Baseline (B)	Occlusion (O)	Recovery (R)	B	O	R
PPG amplitude	Test	434 (37; 781)	13 (6; 63)	509 (173; 736)	0.158	0.002 *	0.084
	*p*-value	-	0.003 *	0.084			
	Contralateral	462 (49; 760)	457 (63; 725)	496 (59; 673)			
	*p*-value	-	0.006 *	0.117			
Alpha exponent	Test	1.55 (1.52; 1.57)	1.44 (1.43; 1.45)	1.54 (1.49; 1.55)	0.394	0.007 *	0.386
	*p*-value	-	0.015 *	0.292			
	Contralateral	1.55 (1.50; 1.58)	1.53 (1.50; 1.57)	1.52 (1.51; 1.58)			
	*p*-value	-	0.269	0.539			
Complexity Index	Test	13.4 (8.0; 19.4)	4.5 (0.0; 12.7)	8.6 (4.0; 12.4)	0.754	0.004 *	0.002 *
	*p*-value	-	0.006 *	0.002 *			
	Contralateral	12.3 (8.4; 21.5)	13.5 (9.1; 26.7)	11.4 (4.8; 20.4)			
	*p*-value	-	0.239	0.308			

## Data Availability

The data presented in this study are available upon request from the corresponding author.
